# Who owns the German subsurface? Ownership and sustainable governance of the subsurface in Germany

**DOI:** 10.1007/s10668-021-01530-w

**Published:** 2021-07-17

**Authors:** Frederic Berger, Philipp Blum

**Affiliations:** Karlsruhe Institute of Technology (KIT), Institute of Applied Geosciences (AGW), Kaiserstraße 12, 76131 Karlsruhe, Germany

**Keywords:** Royalties, Subsurface governance, Energy policy, Sustainability, Mining law

## Abstract

In 1980, the Federal Mining Act was introduced to govern the use of the German subsurface. By paying royalties, companies can get permission to exploit resources. Yet, there is no official report breaking down the payments for hydrocarbons and lignite, in particular regarding the effectively levied fees. Hence, the objective of this study is to provide an overview of the ownership and paid royalties, and to discuss the sustainable use and management of the German subsurface in the face of ecological, social, and economic impacts of resource exploitation. Our analysis shows that the subsurface is partly state- and partly company-owned. Lignite is almost exclusively privately owned by two companies. In contrast, hydrocarbons are predominantly state-owned. In 2017, on average 13% was paid in royalties for gas and 11% for petroleum. These royalties have minor impact on state budgets. For instance, in the concerned state of Lower Saxony, the levies amount to 189 million € or 0.6% of the state budget. Thus, the state income from royalties is low. However, local communities and property owners have no financial benefits. Finally, to obtain a more sustainable use of subsurface, the current Federal Mining Act must be adapted to account for environmental and social impacts.

## Introduction

Due to the increase in global population and the resulting increasing demand of energy and natural resources, there is an urgent need for sustainable use and management of the subsurface and its resources (Jerneck et al. 2011). For instance, Velis et al. (2017) have investigated the sustainable use of groundwater resources and concluded that a sound understanding of local groundwater characteristics and human impacts is crucial to implement sustainable goals for this water resource. Furthermore, as urbanisation advances globally, sustainable development and management become even more crucial in urban areas (Bobylev 2009).

Hence, for a sustainable use and management of the subsurface and its resources, it is paramount to understand the subsurface’s ownership structures. According to the Extractive Industries Transparency Initiative (EITI, a worldwide initiative for transparency in the resource extraction sector featuring 53 national offshoots), Germany is one of the most transparent countries, when it comes to the exploitation of subsurface resources (EITI 2020). Yet, there is no official report breaking down the payments for hydrocarbons and lignite, in particular regarding the effectively levied fees. In our study, we therefore focus on the legal ownership of the subsurface in Germany and the royalties paid for the use of natural resources, such as gas, petroleum, and lignite. The latter generates the overwhelming majority of these levies in Germany.

Khelil (1995) has categorised royalties alongside taxes and other levies as a part of a superordinate fiscal regime, setting the “price” or government’s take for the exploitation of natural resources. This regime is then compared by private actors alongside local geology and other costs, in order to assess potential profits. Supporting this framework, Blake and Roberts (2006) have undertaken a comparison, analysing jurisdictions’ fiscal regimes in Canada, Papua New Guinea, Sao Tome and Principe/Nigeria Joint Development Zone, Tanzania, and Trinidad and Tobago. Discovering distortionary effects reducing the profit margins for higher production rates, the reduced incentives for higher reservoir recoveries for companies are criticised. Country-specific assessments of fiscal regimes have also been undertaken, for instance for low-grade hydrocarbons in China (Cui et al. 2011). The latter research has called for stronger incentives for private actors to exploit less profitable petroleum and gas fields in China, proposing to merge the different resource levies and reducing them to a range of 1% to 10%. However, these publications consider hydrocarbons as a pure economic good, without taking social and environmental implications into account. Consequently, the underlying objective is an extensive exploitation of the resources by increasing the investment attractivity for private actors, ignoring potential negative societal impacts.

In his analysis of the South African mineral and petroleum resources royalty act, Cawood (2010) has also taken the societal significance of royalties into account. Furthermore, he has discussed the question, which level of government should be entitled to levy them and how they should subsequently be distributed. Plourde (2010) has conducted a similar study for the royalties on oil sands in Alberta, Canada. He has concluded that the role of the federal government as a fiscal player in oil sands development declined over time, inter alia, risking to finally pay a greater proportion for social and environmental consequences.

Currently, Germany is addressing an energy transition from fossil and nuclear to renewable energy sources. Thus, the use and management of the subsurface play an important role not only as a natural energy source for fossil energy, such as lignite and hydrocarbons, but also as a thermal, electrical, and material storage for natural gas, methane, hydrogen, carbon dioxide, and nuclear waste (Kabuth et al. 2016). According to the Federal Mining Act (Bundesberggesetz, BBergG), the use of the deeper subsurface in Germany is currently managed on the basis of ’first-come first-serve’, in which the first applicant is granted exclusive prospection and utilisation rights.

**Fig. 1:**
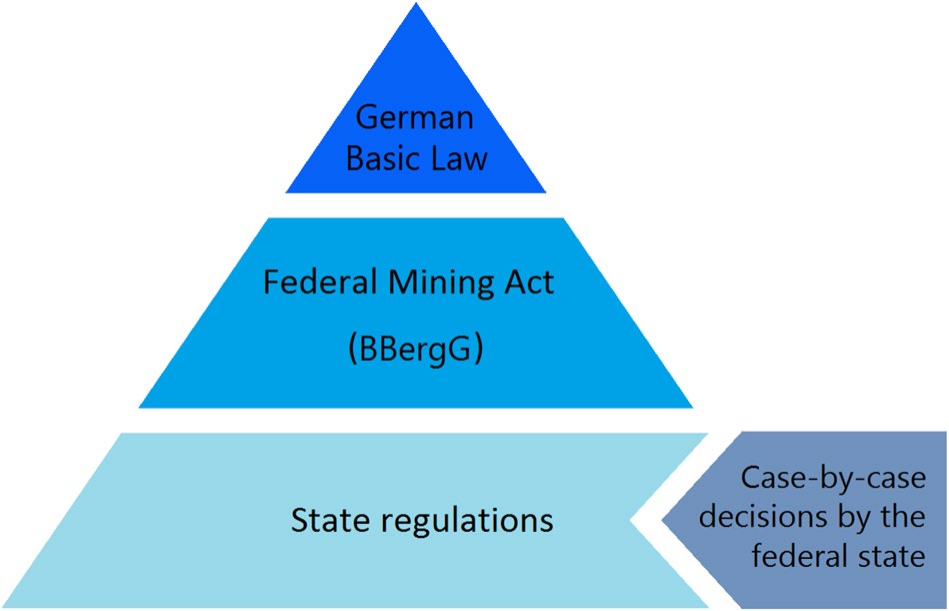
Hierarchy of rules of the German mining legislation

The Federal Mining Act (BBergG) was passed by the German parliament in August 1980, replacing a variety of previous regional mining laws (Fig. 1). This law subdivides the ownership of the subsurface’s assets into freehold and freely mineable resources. With the possession of the ground comes the right of mining for the freehold resources. However, freely mineable resources, constituting the majority of subsoil assets, belong to the states (Fig. 1). Their rights of mining must be granted by the responsible mining authority of the states. Hence, the federal states have the possibility to levy royalties on exploited freely mineable resources.

In general, the BBergG defines a standard royalty rate of 10%, but allows the states to adapt those rates within a range of 0% to 40%, as well as individually for each resource. A different legislation applies to resources subjected to so-called Old Rights. Those were granted before the inauguration of the Federal Mining Act and can therefore, following the superordinate German Basic Law, not be withdrawn (Fig. 1). Furthermore, resources subjected to Old Rights are exempted from royalties.

In the last decades, following the rise of the environmental movement, public actors and various stakeholders have complained about the Federal Mining Act being obsolete with regard to social and environmental aspects, since it was mainly conceived as a law assuring the raw materials supply. Those actors, including politicians, scientists, environmental groups, lawyers, and even the Federal Environmental Agency (UBA), criticise the lack of sustainability of the German Federal Mining Act, e.g. Penn-Bressel and Weber (2014), Hiß (2015), Beckmann (2015).

The Brundtland Report defines a sustainable development as a “development that meets the needs of the present without compromising the ability of future generations to meet their own needs”. Furthermore, the report calls for an equivalent consideration of ecological, social, and economic aspects. Passet (1979) has specified this definition by defining the social, ecological, and economic spheres as interdependent. Thus, a development is only genuinely sustainable, if those three spheres are individually sustainable and their concerns fully respected (Brundtland 1987). Based on this definition of sustainability, the question whether the German mining policy sustainably manages the subsurface in favour of its citizens and the future needs are also discussed. Hence, the exploitation of German gas, petroleum, and lignite resources is specifically examined regarding the equilibrium between the three spheres.

The ecological sustainability mainly consists of the impacts on the environment. The effects on nature and the groundwater, as well as the emission of toxic gases and carbon dioxide, constitute the main-related aspects. The social sphere concerns the impacts on the people and communities. The associated major facets are the influence on people’s health, the effect on employment, and in the case of lignite, the relocation of inhabitants for opencast mining. However, currently only the economic sphere is thoroughly considered by the prospecting and exploring companies. Hence, a holistic and sustainable view of the subsurface is still lacking and therefore also discussed in this study.

## Research methodology

This study primarily focuses on the royalties for hydrocarbons and lignite, whereby only in East Germany for the latter. This follows from the fact that the lignite resources in West Germany were already sold to companies long before the Federal Mining Act’s adoption (Kölnische Rundschau 1950). Hence, the Old Rights had to be acknowledged within the new legislation. This is not the case for the East German lignite, which was mainly privatised within the reunification of 1990 and therefore a decade after the introduction of the Federal Mining Act (Schwenn 1994; DPA 1993).

The data originate from various sources. It has been gathered through a review of relevant reports, informal interviews, and inquiries directed to the mining authorities.

### Review of relevant reports

The main source of information for this research has been a variety of reports issued by public bodies, non-governmental organisations, trade associations, and companies.

The details about the paid royalties on petroleum and gas are taken from the 2018 report of the BVEG, the German petroleum and gas trade association (BVEG 2019). The report includes data voluntarily provided by various exploration and production (E&P) companies operating in Germany, enabling first estimates of the paid royalties. The accuracy of the data is not proven, since those statements are not compulsory or in any way legally binding. According to an employee of the BVEG, the numbers are rounded by the companies and are only superficially reviewed by the trade association. However, this report is the only source subdividing the payments to the states into levies for petroleum and gas. Furthermore, the BVEG normalises the exploited gas volumes to a calorific value of $CV = 9.7692$ kWh/m$^3$, allowing a better assessment of the monetary value with the gas prices issued in €/kWh by the Federal Statistical Office of Germany (Destatis 2020b).

The paid acquisition prices for the East German lignite are part of business contracts and therefore kept under lock, with the archival thirty-year period expiring in the coming years. A 2013 parliamentary request by the Green Party in order to find out the buying prices of those lignite districts was denied due to contractual secrecy (Deutscher Bundestag 2013). Nevertheless, circulated paid sums exist in contemporary newspaper articles and press agency reports (DPA 1993; Schwenn 1994).

### Informal interviews

In order to classify the gathered information, many informal phone calls and email exchanges have been carried out. For instance, conversations with the Federal Environmental Agency (UBA) have been used to locate general and sustainability issues within the German Mining legislation.

Exchanges with the majority of the state authorities for mining have been valuable to clarify the different regional regulations for hydrocarbons and their realisation. To take the industry’s perspective into account, phone interviews with E&P-companies and trade associations have been conducted.

Regarding lignite, communication with the Federal Institute for Geosciences and Natural Resources (BGR) has been useful to clarify the legislative framework of the Old Rights. Several exchanges with parliamentarians and their consultants have been conducted to include the problems originating from the extraction of lignite.

### Inquiries to the mining authorities

In order to receive improved data for the royalties, we have posed an inquiry to the Lower Saxony State Authority for Mining, Energy, and Geology (LBEG) following the Environment Information Act, which is derived from the Aarhus Convention (UNECE 2014). It allows the demand of available environmental information and obligates the concerned authority to hand over its data as fast as possible and normally within a month (BMJV 2017). We have requested the available data about the petroleum and gas fields in Lower Saxony, Schleswig-Holstein, Hamburg, and Bremen, especially the paid royalties, potential abatements, the offset on-site treatment costs, and the oil grade for the petroleum, to assess its value. We have asked to categorise the data into exploitation fields and years for the period from 2016 to 2018, sending the request on the 30th of July 2019. After consulting the relevant companies, the LBEG has denied the inquiry on the 30th of October 2019 on the ground that the requested data do not constitute environmental information and the access to this information is outweighed by the protection of company secrets. However, one company making up for 0.3% of the petroleum and 0.04% of the gas production in Germany voluntarily has provided the requested information, thereby helping us to define the oil grade in the main petroleum area in the western part of Lower Saxony (BVEG 2019).

In order to eradicate the conflict arising through the protection of business secrets, we have posed a modified inquiry on the 15th of November 2019. We have requested the same information as in our first inquiry but grouped exploitation fields with the same royalty rates. This would have made it nearly impossible to allocate the paid royalties to specific fields and therefore to companies. In a phone call, an employee of the LBEG has explained that there was low prospect for our request since the demanded data still do not constitute environmental information in their opinion. Hence, we have finally decided to withdraw our second request on the 18th of December 2019.

### Data assessment and processing

By comparing the data based on the BVEG reports (BVEG 2019) and on the second report published by the German EITI offshoot (D-EITI 2019), the accuracy of the former’s figures can be assessed. Given the fact that the D-EITI report does not categorise the states’ royalties into the different subsoil assets, the comparison can only be made for states receiving no levies but for hydrocarbons. This is the case for Schleswig-Holstein (2016, 2017), Hamburg (2016, 2017), and Bavaria (2017), where the stated royalties in the BVEG report are between 1% and 10% above those stated by the D-EITI report. This divergence amounts to an average of 6.1% and is systematic.

In the case of the East German lignite, the buying prices were paid in German mark (DM) in 1993 and 1994. Hence, to be able to compare them to hypothetically owed royalties, the inflation over the past three decades (issued by the Federal Statistical Office, (Destatis 2020c)) must be considered for both the buying prices and the levies. The complete calculation path of the theoretically due royalties is described in "Appendix A1 and A2".

**Table 1:** Legislative framework regarding royalties for petroleum and gas in the federal states

Federal state	Royalty percentage		Based on state royalty regulation
	Petroleum	Gas	
Baden-Württemberg	19^a^	37^a^	Yes
Bavaria	0^b^	0^a^	Yes
Berlin and Brandenburg	1^a^	10^a^	Yes
Bremen	10	10	No
Hamburg	7^a^	37^a^	Yes
Hesse	10	10	No
Lower saxony	0^a,d,e^	27^a,d,e^	Yes
Mecklenburg-Western Pomerania	10	10	No
North Rhine-Westphalia	10	10^a,e^	For gas
Rhineland-Palatinate	12^a,e,f^	10^f,g^	For petroleum
Saarland	10	10^a,h^	For gas
Saxony	10	10	No
Saxony-Anhalt	10	10	No
Schleswig-Holstein	40^a,i,^	40^a,i^	Yes
Thuringia	10	10	No

$^{\rm a}$ Deduction of on-site treatment costs

$^{\rm b}$ 5% on petroleum from the field Aitingen. Deduction of a flat fee of 25 €/tonne for on-site treatments

$^{\rm c}$ Royalty for Breitbrunn-Eggstätt in form of a one-time payment of 300 000 DM

$^{\rm d}$ Petroleum and associated gas excepted from Rühlermoor Valendis, Bramberge Emlichheim, and Georgsdorf are free of charge

$^{\rm e}$ Reduced rates for reservoirs more difficult to exploit

$^{\rm f}$ 15% on petroleum from Römerberg, 7% from Rülzheim

$^{\rm g}$ Associated gas directly converted into electricity is free of charge

$^{\rm h}$ Gas from the field Saarbrücken-Süd is free of charge

$^{\rm i}$ For the fields Nordsee A6/B4 and Heide-Mittelplatte I the rate for gas is 18% and between 21% and 40% for petroleum, depending on the current market value of petroleum (Fig. 4)

## Results

### Petroleum and gas

The vast majority of the German petroleum and gas is subjected to the Federal Mining Act. This means, the resources are owned by the federal state and companies can only be granted rights of mining. In return, the federal states levy royalties. While in former West Germany, only few hydrocarbon sources are subjected to Old Rights, nearly all resources in the former East German states are company-owned. They were sold as resources subjected to Old Rights in the course of the reunification.

**Fig. 2:**
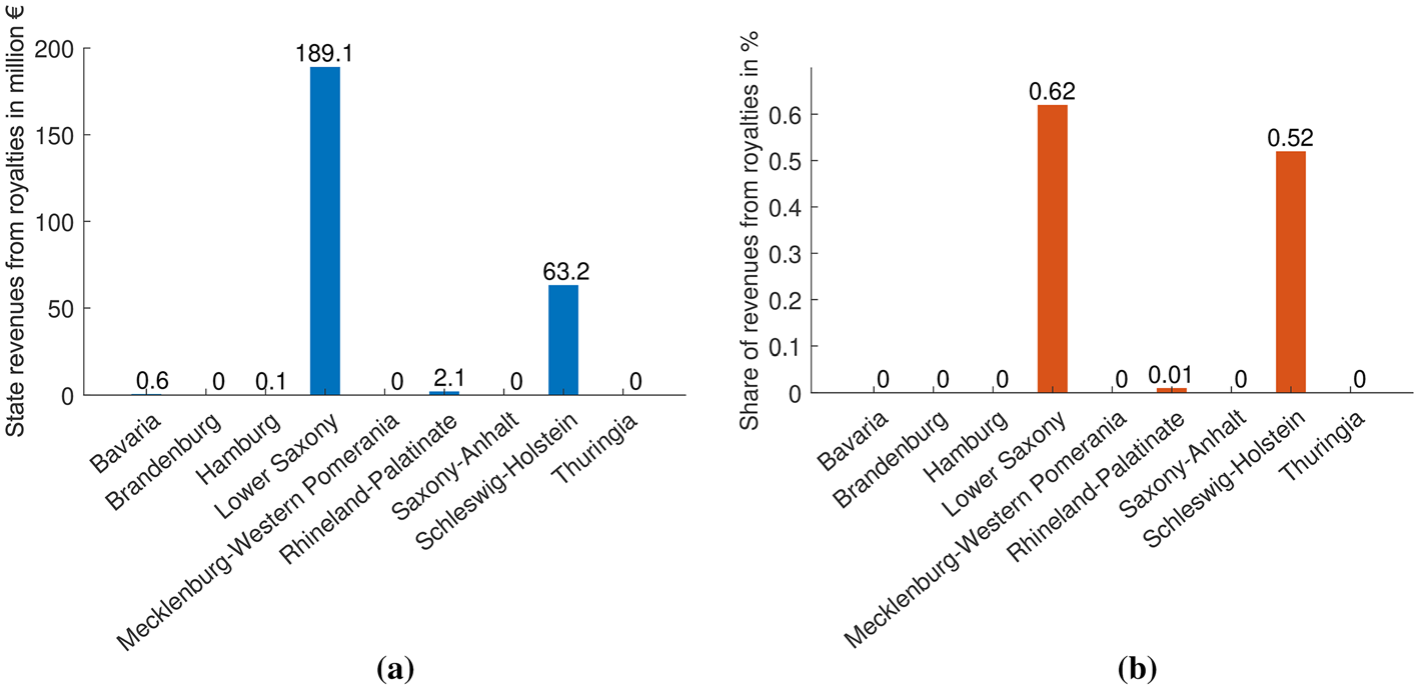
Paid royalties in the nine states with hydrocarbon exploitation (**a**) and share of their total state revenues originating from royalties (**b**) in 2017 *(Data source:* BVEG (2019))

**Fig. 3:**
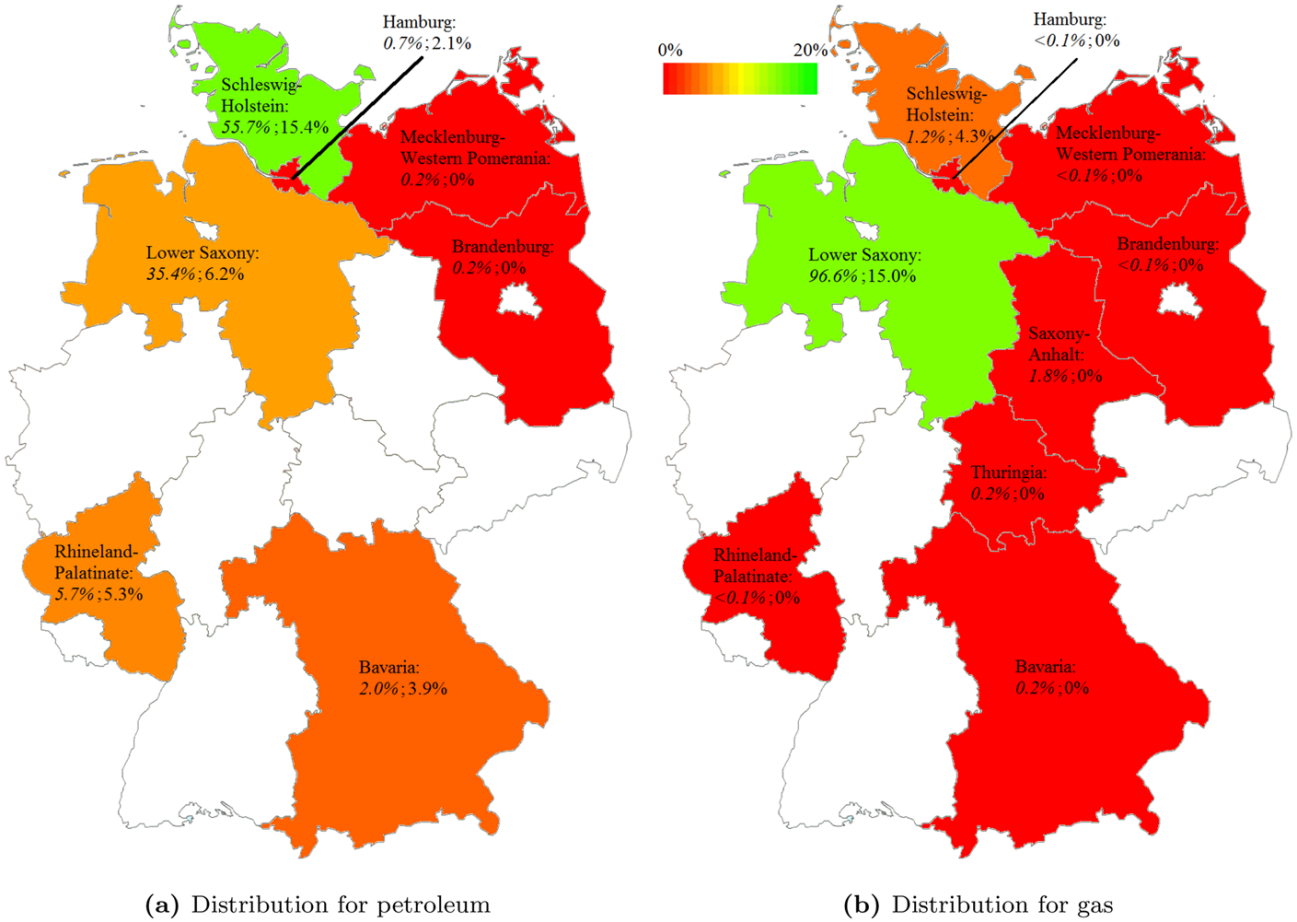
Proportion of the production (*italic*) and effectively paid royalty rates (upright) for petroleum (**a**) and gas (**b**) in the German states *(Data source:* BVEG (2019))

Due to state regulations, the royalty rates differ from state to state (Table 1; Figs. 2, 3). Some states, mostly without revenues from royalties, rely only on the Federal Mining Act for hydrocarbons. Since it can be used to regulate the exploitation of resources, the governance varies throughout the other states. In recent years, Bavaria dropped the rate for hydrocarbons to zero, in order to create incentives to explore. Because of its inefficiency, the decision for this strategy was later withdrawn and a return to the BBergG’s rates is envisaged. In Lower Saxony, every petroleum source with an annual exploitation below 30 000 t is exempted. Throughout Germany, only seven sources produce annually more than 30 000 t, with four sources in Lower Saxony only. The state also gives reductions on the royalty rates ranging between 50% and 75% for reservoirs more difficult to exploit.

**Fig. 4:**
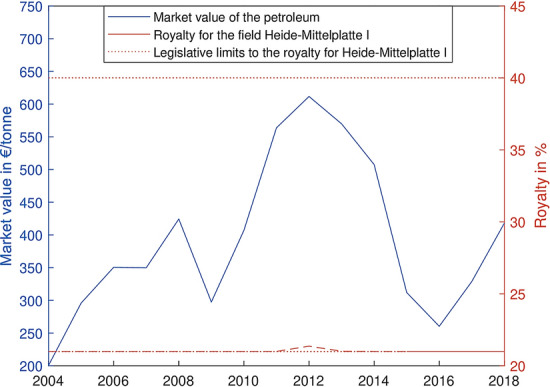
Royalty on petroleum from the biggest German field Heide-Mittelplatte I, since 2015 depending on the current petroleum price following (Eq. 1) *(Data source:*LBEG (2019b))

In 2015, Schleswig-Holstein adopted a new regulation raising the royalty rates to the maximal rate of 40%. In order to reduce the impairment for running exploitations, a special formula was therefore implemented. The latter in particular addresses the largest petroleum field Heide-Mittelplatte I, producing 55% of Germany’s petroleum, and the only offshore gas field Deutsche-Nordsee A6/B4. While the royalty on gas amounts to 18%, the royalty rate on petroleum is defined by the following second-order binomial equation, considering the current petroleum market value:

1 $$\begin{aligned} R = 0,00013851 \cdot P^2 - 0,15525 \cdot P + 64,5 \end{aligned}$$

with *R* as the royalty in percent and *P* as the petroleum price in Euro per ton. Furthermore, the regulation defines 21% and 40% as minimal and maximal royalty rate, meaning that for every petroleum price below 555,56 Euro per ton, the rate is set to 21%. When prospected onto the last fifteen years, the royalty rate never reached 22%, even though there were significant petroleum price fluctuations (Fig. 4).

Hence, the paid royalty rate lies below the regulations’ standard rate in every state (Table 1, Fig. 3). This is caused by reductions for special sources and, more importantly, on-site treatment costs. The costs accruing from the on-site preparation of the resource can be offset from the initial payment. That is the main reason for the divergence between the rates provided in Table 1 and in Fig. 3. Significant revenues are only levied in three states, 189.1 million € in Lower Saxony mainly through royalties on gas, 63.2 million € in Schleswig-Holstein and 2.1 million € in Rhineland-Palatinate from petroleum (Fig. 2a).

Nevertheless, there is a sub-linear correlation between the proportion of the national resource production and the state-specific royalty rates. Federal states with a larger production rate tend to levy higher effective royalty rates (Fig. 3).

### Lignite

**Fig. 5:**
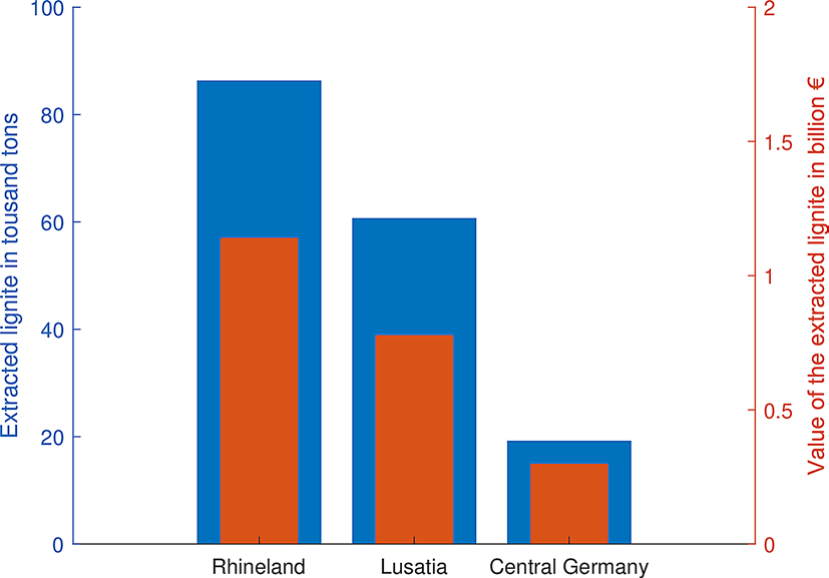
Amount and value of the extracted lignite from the three lignite districts Rhineland (RWE), Lusatia (LAUBAG) and Central Germany (MIBRAG) in 2018 ("Appendix A1") *(Data source:*Statistik der Braunkohle (2019))

Legally, even though it constitutes a freely mineable resource, the vast majority of the German lignite is owned by private companies. There are three major lignite companies operating in Germany, RWE Power AG (RWE) in the Rhineland basin, MIBRAG in the Middle German basin, and LAUBAG in the Lusatian basin (Fig. 5). While the first company belongs to the RWE corporate group, MIBRAG and LAUBAG belong to a Czech private equity firm with a focus on legacy energy sources. This company called Energetický a Prűmyslový Holding (EPH) is known for owning Europe’s most climate-damaging energy provider EP Power Europe (Smid 2017).

The ownership of the three main mining areas has different origins. The Rhineland basin has been owned by RWE since 1914, when it started its electricity production from lignite (Klein 1999). In contrast, the lignite in the Middle German and Lusatian basins was sold in the course of the reunification in 1993 and 1994, respectively (DPA 1993; Schwenn 1994). Thus, it was sold more than a decade after the Federal Mining Act was adopted. Even though the new legislation did not offer any legal way for it, the lignite was sold as resource subjected to Old Rights.

Beside the privately owned areas, only smaller districts owned by the states of Saxony and Saxony-Anhalt remain. Hence, following the Federal Mining Act they would be subjected to royalties, however, they are not levied. In Saxony, the state’s regulation explicitly exempts lignite from this levy, thereby potentially missing out on approximately 265 million € (Kuhr 2016). Platter (2008) from Brandenburg’s parliament advisory service has authored a report, concluding that levying royalties on state-owned lignite would constitute an unequal treatment, given the fact that company-owned lignite is exempted. Hence, no royalties are levied on lignite.

For the two private companies, circulated purchasing prices exist. The MIBRAG was sold in December 1993 for 2 billion DM, including a 41.1% share of the planned power plant Schkopau, worth 2.7 billion DM (DPA 1993; Reuters 1993). The LAUBAG was sold in September 1994 for 2.1 billion DM (Schwenn 1994). Subtracting the share of the power plant and adjusting for inflation gives acquisition prices in 2020 of 0.64 billion € for the Middle German and 1.51 billion € for the Lusatian basin.

**Fig. 6:**
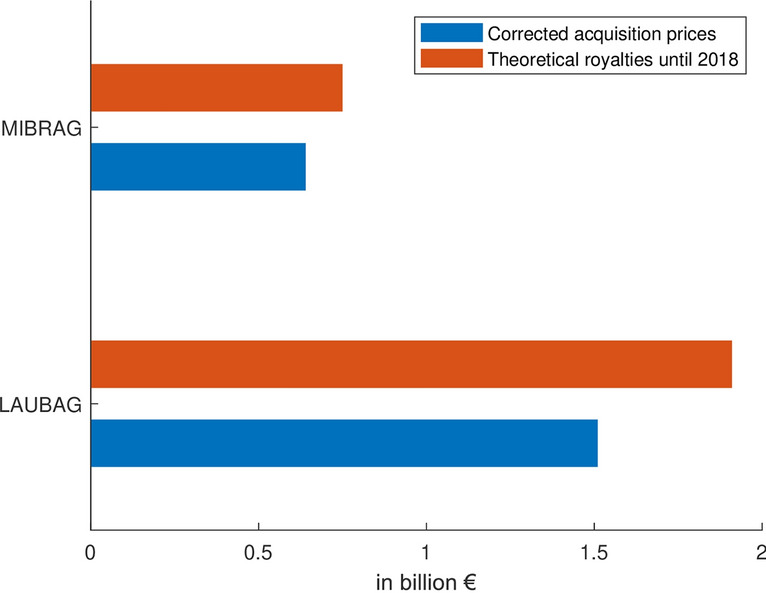
Comparison of the acquisition prices and a hypothetical royalty of 10% derived from the Federal Mining Act for the two coal-mining districts in East Germany, the Lusatian basin (LAUBAG) and the Middle German basin (MIBRAG) ("Appendix A1 and A2")

Figure 6 compares the prices to theoretically paid royalties (BBergG standard rate of 10%) until the year of 2018. It shows that already in 2018, the latter would have been significantly higher than the paid acquisition prices. In addition, the paid sums not only include the rights of mining but whole running companies, with assets such as buildings, machines, liquidity. It is to be assumed that the lignite companies were sold below value, which was not an exception for privatisations during the reunification, as Bleicher (2007) states for the sale of the utility companies.

## Discussion

### Petroleum and gas

Germany covers 58.4% of its primary energy needs with hydrocarbons, 34.9% with petroleum and 23.4% with gas (BP 2019). However, only a fraction originates from domestic sources. Given the fact that petroleum and gas are freely tradable resources, the German hydrocarbon combustion is independent from the domestic production. Reinforcing this effect, the German gas network will change from low caloric gas (L-Gas, mainly from Germany and the Netherlands) to high caloric gas (H-Gas, mainly from Russia and Norway) in 2030. Thus, in the future, it will no longer be able to transport its own domestic gas (Kandzorra 2020).

**Fig. 7:**
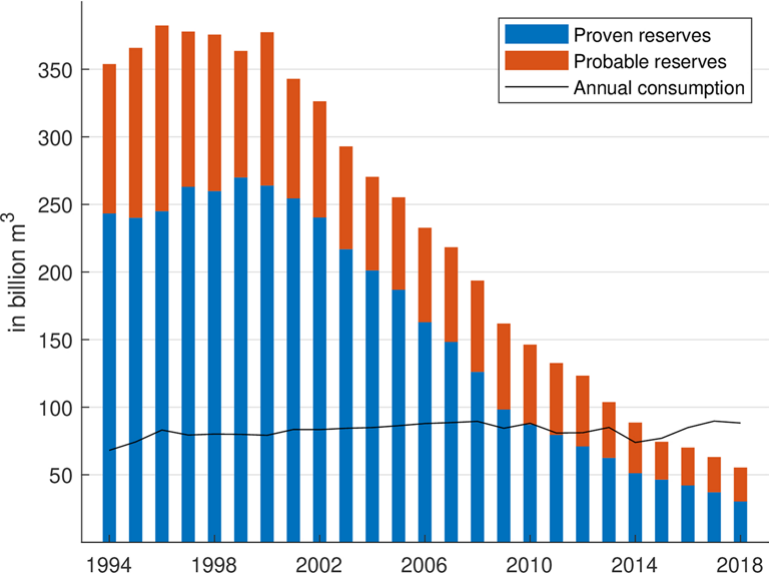
Development of the proven and probable gas reserves compared to the annual gas consumption in Germany *(Data sources:* LBEG (1996-2019); BP (2019))

**Fig. 8:**
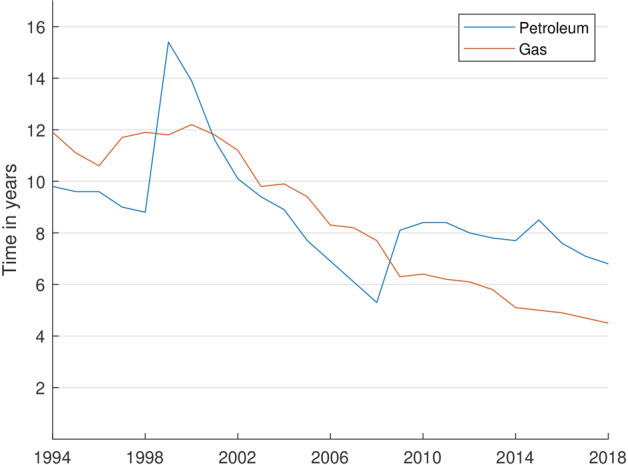
Development of the statistical range of coverage (quotient of reserves and extraction rate) of proven petroleum and gas reserves in Germany *(Data source:* LBEG (1996-2019))

During the past decades, the reserves have massively declined. In 2000, the probable and proven gas reserves were about seven times greater than in 2018 (Fig. 7). The statistical range of coverage, showing the period the reserves last under the current production rate, has also drastically decreased. It amounted to less than seven years for petroleum and less than five years for gas in 2018 (Fig. 8). This is mainly due to the unsustainability of the Federal Mining Act. In §1 of the BBergG for instance, it is stated that the main aim of the law is to ensure the mineral supply. Hence, the mining authorities hardly have any decision-making scope for a denial of an application, if formalities of the application are satisfied (Kabuth et al. 2016).

A benefit of the exploitation of hydrocarbons in comparison to other sources of energy is the low space requirement. In order to exploit entire reservoirs, spaces below a hectare can be sufficient, reducing the impact on the surface environment. The main ecological issues are possible leakages (Davies et al. 2014). The transported liquids, the resource, and the reservoir water contain toxic substances such as mercury, benzene, or xylene (Schomann 2017). While the petroleum leakages mainly cause soil contaminations, gas extraction also causes methane leakage. According to Howarth (2015), the average leakage for conventional gas extraction amounts to 4%. This has a significant impact on climate change, given the fact that methane is an about 86 times more harmful greenhouse gas than CO$_2$. With regard to the greenhouse effect, this makes gas a more polluting source of energy than hard coal and petroleum (Howarth 2015).

By granting royalty reductions, the legislation provides companies with false incentives. In Lower Saxony for instance, the state with the highest revenues from royalties, three reductions’ effects are detrimental to the environment. For petroleum sources exploited with tertiary recovery methods, the original royalty rate is halved. Such methods consist of injecting fluids into the reservoir in order to increase the pressure and the fluid viscosity. However, this can induce seismicity, as it occurred in Lower Saxony (Schomann 2017). Tertiary recovery methods are also used to exploit gas from reservoirs with permeabilities below 0.6 millidarcy, which reduces the royalty rate by 75% for the first five years of the extraction. The state also reduces the rate by 40% for reservoirs with extraction rates below 4 500 m$^3$/h. The latter applies to more than half of the state’s sources (LBEG 2019a). However, as Balcombe et al. (2017) have shown, these small onshore reservoirs constitute an above-average portion of methane leakages from conservative gas sources. Until today, the tertiary recovery methods in Germany mainly consist of hot water and steam injection, while the addition of chemicals is still uncommon.

**Fig. 9:**
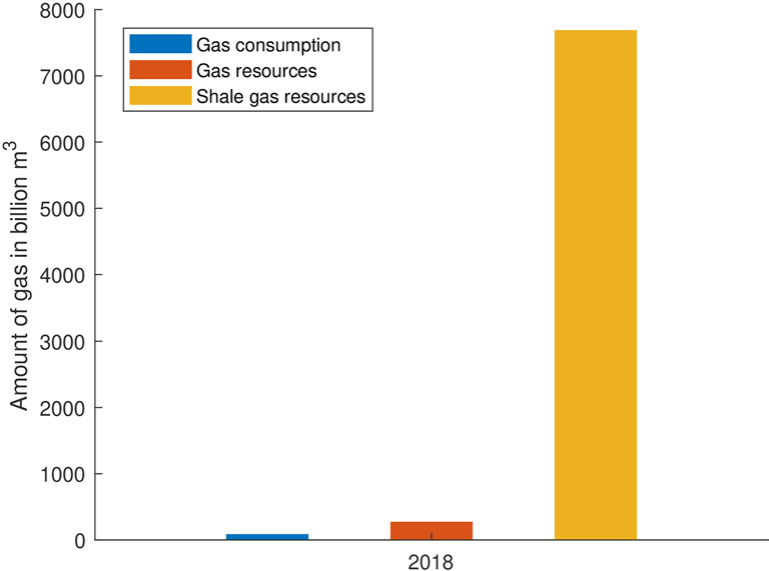
Comparison of the annual gas consumption, the conventional gas resources, and the shale gas resources in Germany *(Data sources:* BP (2019); LBEG (2019a); BGR (2016))

In the face of the rising potential of German shale gas (Fig. 9), the current legislation has not been keeping up. Instead of banning or regulating fracking because it violates the precautionary principle, the government simply raised the hurdle by making small changes to the Water Protection Act (Engelhardt and Louis 2014). This caused Schleswig-Holstein to raise the royalties for hydrocarbons to the maximum rate to reduce the incentives for the cost-intensive fracking technology. In contrast, Lower Saxony has had to grant claims for fracking, fearing actions for failure to act from the E&P-companies (Wetzel 2016). Consequently, there is an urgent need to revise the law by including sustainability principles into the Federal Mining Act (BBergG).

In 2018, approximately 3000 people worked in the petroleum and gas resource sector. They are mostly based in Lower Saxony. Recently, it was disclosed that the extraction of hydrocarbons has negative impacts on the local population. In Rotenburg (Wümme), one of the main gas regions, the leukaemia case numbers for men have been twice as high as usual. In addition, other forms of cancer have occurred more frequently. Hence, families are concerned about their health (Betzholz and George 2016). This matches the results from previous research in North America, suggesting a correlation between the distance to gas wells and health impacts (McKenzie et al. 2014; Kassotis et al. 2014; Stacy et al. 2015).

The royalties have an impact of more than 0.1% of the budget in only two states. In Lower Saxony, they amount to 189.1 million €, and 0.62% of the state budget, which is roughly the equivalent of the housing and urban development expenses (MFNds 2017). In Schleswig-Holstein, the royalties’ share of the budget is 63.2 million € and 0.52% of the state budget, which is comparable to three quarters of the expenses for housing and urban development (FMSH 2017). For those payments, the companies provide 2.0% of the domestic petroleum and 6.4% of the gas needs (LBEG 2019a). According to the German petroleum and gas trade association (BVEG), these supplies make Germany less dependent on foreign resources. However, even if the autarky is increased during the time of production, the dependency from other countries grows as the resource is constantly depleted. While the proven gas reserves in 2000 sufficed to supply Germany for three years, the reserves in 2018 could only cover a third of the domestic consumption (Fig. 7). In the case of a crisis, Germany barely holding any backup reserves would be at their suppliers’ mercy. The latter is a very critical aspect, as we can currently also observe during the coronavirus pandemic (COVID-19), in which countries are unable to match their own demand on protective clothing and masks. Thus, the aspect of independence of hydrocarbon imports in form of a strategic national reserve should be seriously considered for the future management of domestic resources.

### Lignite

Through the company-ownership of the German lignite, the federal states have hardly any governance scope on the resource. Enforcing sustainable policies is therefore nearly impossible through state regulations. Even though a shortage of this resource is not to be expected in the next 200 years, a lot of other issues play a role concerning the sustainability of lignite extraction (BP 2019). It is also important to assess the combustion of lignite, since it can only be used nationally. The high water and low energy content make transport unprofitable, especially compared to hard coal.

Additionally, lignite combustion massively pollutes the air (Wronski and Sorge 2015). Even though it contributed to less than 24% of the German electricity production in 2015, it emitted 45% of the carbon dioxide, 44% of the lead, 53% of the sulphur, 62% of the nitrous oxide, and 67% of the mercury emissions of the energy sector, among several other toxic emissions. Furthermore, it has massive impacts on water quality and groundwater levels (Drosihn et al. 2017). The recultivated areas often take years to redevelop a natural flora and fauna. Only after 40 years of recultivation, the soils recover their initial agricultural quality (Wronski and Sorge 2015).

One of the main arguments for the continuation of lignite mining is the related employments. In January 2020, a total of 12 084 people was working in the lignite sector in Germany (BMWi 2019). A small amount of those people works directly in lignite extraction, with potentially poor prospects in the current and future labour market. Although, according to the European Parliamentarian Michael Bloss, the German state railway submitted takeover offers for many of those workers in the case of complete mine closures. Furthermore, lignite mining and combustion have negative impacts on the population, not only locally but also beyond the related regions. In order to make way for lignite exploitation, which is done in opencast mines in Germany, inhabitants are relocated, and villages demolished. More than a 100 000 people within 90 years have lost their homes to opencast mining and more are expected to be relocated in the next decades (Wronski and Sorge 2015). In addition, Jensen et al. (2013) calculated from governmental data that the German coal combustion annually costs 29 271 years of lifetime or 2 722 fatalities. The combustion of lignite contributes the major share to this figure.

**Fig. 10:**
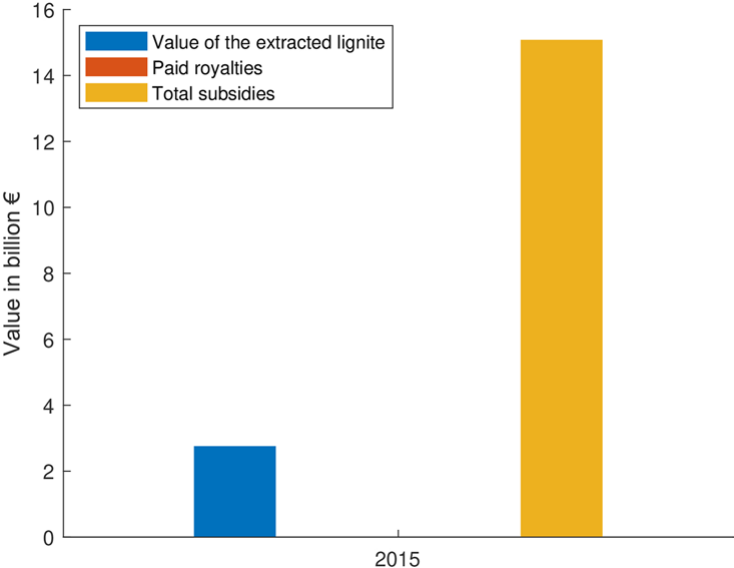
Value of the annually extracted lignite, the paid royalties, and the total subsidies for lignite in 2015 ("Appendix A1") *(Data source:* (Wronski and Sorge 2015))

Due to the sale of the companies in the nineties, the federal government obtained large sums. This money compensated in part the federal program restructuring the former East German companies before their privatisation. In addition, the lignite production increases the German self-sufficiency and renders the German energy supply less market dependent. Furthermore, coal-based electricity is the cheapest available in Germany, far below the price of energy from renewable sources. This is not only due to the cheap resource, but mainly to a variety of subsidies. Those surpass by far the value of the mined lignite, as shown in Fig. 10 (Wronski and Sorge 2015). However, Rehbock and Kolbe (2013) show that the electricity price has very little impact on the economic power. Higher energy prices actually increase companies’ resilience to price variations and make them significantly more energy efficient.

Unfortunately, the continuation of lignite combustion will also cost Germany large amounts of compensation payments. The Paris Agreement specifies that a CO$_2$-surplus must be compensated through the European emissions trading system (Höhne and Fekete 2019). According to the federal government’s 2019 projection report and climate protection plan, missing the target will cost between 25.8 and 27.8 billion € for the energy sector alone (BMU 2016, 2019) ("Appendix A3"). Lignite contributes to almost half of these costs.

Moreover, the ability to cover the costs of recultivation seems uncertain for East Germany’s lignite mining districts. The purchase of MIBRAG was massively financed from its own recultivation fund, whilst in the following years, significant profit transfers towards EPH took place. In total, those transactions summed up to more than 400 million €. Meanwhile, the recultivation fund contains merely about 200 million € and experts estimate that this amount will be insufficient for necessary future recultivation works (Landtag Brandenburg 2017). In 2016, the Swedish state company Vattenfall sold LAUBAG without purchase price to EPH (Berger 2016). Included were 15 billion Swedish kronor (SEK) (1.34 billion €) in cash and 18 billion SEK (1.61 billion €) in the recultivation fund (Vattenfall 2016). Greenpeace and a Mongolian investment group were excluded from the sale without reasons, even though the latter was willing to pay up to 1.85 billion €. This exclusion led to an inquiry by the European competition authority (Iwersen 2016). Afterwards, Smid (2017) found out that the sale to EPH was arranged by the Ministry of Energy of Brandenburg before Vattenfall publicly announced their willingness to dispose of LAUBAG. A month after the acquisition, one of the owners and the main financier of the deal was sold out of the holding for 3.25 billion €, which Smid (2017) assumes was done with money acquired from Vattenfall. By now, the recultivation fund holds about half of the required 3 billion € and the company has been making triple-digit million losses in the recent years. Thus, there is currently no fulfilment of LAUBAG’s recultivation obligations in sight (Landtag Brandenburg 2017; Reiß and Harms 2018). Furthermore, the operative business is managed by a limited company. The ties to the owners include numerous shell companies, among others in the well-known tax havens of Luxembourg and Cyprus (Smid 2017). It is not clear yet, whether EPH would be liable for an insolvency of the operational limited company in the face of the recultivation costs (Reiß and Harms 2018). Hence, the German taxpayers risk to take the fall in the end and pay for the recultivation.

In 2020, the German government introduced a law to end the combustion of coal by 2038. Thereby, the government planned to compensate the affected companies with more than 4 billion €, with 1.75 billion € to LAUBAG alone. Shortly after, reports surfaced, showing that the company itself previously planned to end the combustion earlier than the new law would require. After announcing to reconsider the compensations, the federal government has now decided to adopt the initial bill (Traufetter 2020; Flauger 2020). Nevertheless, there seems to be an exit for lignite combustion in Germany. In the context of nuclear energy, the Federal Constitutional Court set a precedence by emphasising the social obligation of property in the face of expropriation (Ekardt 2012). This means that compensations are not to be paid, if the expropriated subject harms society. In our opinion, this should also be applicable for lignite exploration and combustion.

## Conclusions and recommendations

This case study attempts to close the information gap regarding the subsurface ownership structures and related royalty payments. Featuring abundant lignite and ordinary hydrocarbons resources, little is known about paid royalties in Germany. The research has revealed that the German subsurface is partly state- and partly company-owned. While lignite in former West Germany belongs to the joint-stock company RWE, the vast majority of hydrocarbons belong to the federal states. In East Germany, nearly all resources were privatised in the course of the reunification and the overwhelming majority of the lignite is in the hands of a private equity firm. The company-owned minerals are free of royalties, and the benefits remain within the mining companies. For the state-owned hydrocarbons, an average of 13% for gas and 11% for petroleum were levied in royalties in 2017. This equals to 173.3 million € for gas and 80.6 million € for petroleum (BVEG 2019). However, in the face of the consequences and triggered follow-up costs, the state’s benefits from all three resources are comparably small, rendering the management of the subsoil assets unsustainable for Germany and its citizens. Thereby, the German subsurface governance fits into the recurring pattern of privatising profits, while socialising losses and costs.

In order to adapt the governance, politicians have taken a first step in the right direction by modifying the financial equalisation system between states. By reducing the weighting of royalties from 100% to a third, states are encouraged to levy higher rates. However, to accomplish a sustainable subsurface governance, further changes are needed. Most importantly, the Federal Mining Act must be adapted, implementing an easier possibility for mining authorities to deny claims for environmental and/or social reasons. The priority of minerals supply in the Federal Mining Act must concede for a sustainable resource management. Discussions regarding those objections have been frequent but never materialised in the form of legislation. The latest foray for a reform of the BBergG by the state of Lower Saxony has been rejected by the Federal Council in November 2020 (Niedersächsischer Landtag 2020). Furthermore, the Federal Supreme Court’s 2013 decision emphasising the social obligation of ownership must be implemented, in order to simplify the uncompensated expropriation of harmful resources. Additionally, on a state level, incentives for environmentally unfriendly exploitation must be abolished. With an implementation of those changes, a sustainable governance of the subsurface could be reached. Thus, the German subsurface would be managed in favour of Germany’s current and future citizens. Finally, this country-specific study should also be performed for other countries, in order to achieve a worldwide sustainable use, management and governance of the Earth’s resources.

## Data Availability

The data and materials supporting the findings of this study are available from the corresponding author upon reasonable request. The data are not publicly available for legal reasons. No code was used during this study.

## Appendix

### A1: Calculation of the lignite market value

This calculation path is used by the Federal Institute for Geosciences and Natural Resources to assess the lignite’s market value:

$$\begin{aligned} V = C \cdot P \cdot H \end{aligned}$$

with *V* (in €/t) as the lignite’s market value, *C* (in €/tonnes coal equivalent (tce)) as the lignite production’s full costs in 2005 (EWI/EEFA 2007), *P* (in %) as the producer price index for lignite normalised to 2005 (Destatis 2020a) and *H* (in tce/t) as the lignite district specific heating value (Niemann-Delius et al. 2009).

### A2: Adjustment of the acquisition prices

The acquisition prices in DM are mentioned in a newspaper article and a press agency report (Schwenn 1994; DPA 1993). The MIBRAG purchase included 41.1% of a new 2.7 billion DM power plant, which is subtracted from the original price (Reuters 1993). The prices are converted to € with the official exchange rate of 1 € = 1.9558 DM and inflation-adjusted with the consumer price index (CPI) (Destatis 2020c). The theoretical royalties are calculated from the extraction rates and are also inflation-adjusted with the CPI (Statistik der Braunkohle 2019; Destatis 2020c). For the year 1994, a fourth of LAUBAG’s 1994 extraction rate has been considered, as it was sold in the course of September. For the same year, the missing information for the producer price index for lignite has been estimated conservatively to 0.95% of 2005’s value (0.98% in 1995).

### A3: Calculation of the costs for not meeting the Paris Agreement’s goals

The calculation is based on Höhne and Fekete (2019), calculating the costs of missing the Paris Agreement’s goals for the transport sector. According to BMU (2016), the energy sector envisions to emit between 175 and 183 megatons of CO$_2$-equivalent (MtCO$_2$-eq) in 2030, compared to 358 MtCO$_2$-eq in 2014. The government’s projection plans with 297.3 MtCO$_2$-eq in 2020, 301.5 MtCO$_2$-eq in 2025 and 267.3 MtCO$_2$-eq in 2030. We adopted a linear interpolation for the emission goals and the projection and calculated the annual CO$_2$-equivalent emission surplus. The period where compensations are due starts in 2021 and every annual surplus is transferred in the next year’s calculation with an 8% surcharge (Höhne and Fekete 2019). The added up surplus has to be compensated with emission certificates in 2030. In the German projection report by BMU (2019), the certificate’s price is assessed to 35€ in 2030, following the EU-Guidelines 2018. This adds up to 25.8 billion € and 27.8 billion € for 183 and 175 MtCO$_2$-eq as goals for 2030, respectively. These are the equations for the costs *C*:

$$\begin{aligned}&C = 35{{\sf C}\!\!\!\!\raise.8pt\hbox{=}} \cdot \sum _{y=2021}^{2030} \left( p(y) - g(y) \right) (1.08)^{2030-y}\\&\text {p(y) =} \left\{ \begin{aligned} 297.3 + 0.84\cdot (y-2020) \text { until 2025} \\ 301.5 -6.84 \cdot (y-2025) \text { as of 2026} \end{aligned} \right. \\&\text {g(y) =} \left\{ \begin{aligned} 297.3 - 11.43\cdot (y-2020) \text { (183 MtCO}_2-\mathrm{eq)} \\ 297.3 - 12.23 \cdot (y-2020) \text { (175 MtCO}_2-\mathrm{eq)} \end{aligned} \right. \end{aligned}$$

with *p*(*y*) as projection and *g*(*y*) as goals.
